# Impact of Policy Reforms on Hospital-at-Home Services: An Interrupted Time Series Analysis of Shanghai

**DOI:** 10.1093/geroni/igaf122.3505

**Published:** 2025-12-31

**Authors:** Sixian Du, Yaqing Liu

**Affiliations:** Huazhong University of Science and Technology, Wuhan, Hubei, China (People’s Republic); Huazhong University of Science and Technology, Hubei, Hubei, China (People’s Republic)

## Abstract

**Background:**

The rapid growth of aging populations and chronic disease prevalence has increased demand for hospital-at-home (HaH) services, which offer home-based alternatives to traditional inpatient care. However, the policy impact on HaH service development in China remains understudied.

**Objective:**

To evaluate the effects of two major policy reforms on HaH services in Shanghai from 2007 to 2022.

**Methods:**

A two-stage interrupted time series (ITS) analysis with a control group was conducted. Data from Shanghai Statistical Yearbooks were used to assess changes in institutional capacity, home-based medical services, and HaH beds across hospitals and community health service centers. Eleven outcome indicators were analyzed.

**Results:**

Following the first policy in 2010, the number of HaH service providers increased significantly, particularly in community health service centers (β1 + β3 + β5 + β7 = 8.200, P < 0.001). HaH beds per 10,000 population also rose (β1 + β3 + β5 + β7 = 0.406, P < 0.001). However, home-based medical visits declined after both policies (β1 + β3 + β5 + β7 = −90,214.350, P < 0.01), likely due to workforce shortages and service delivery risks. After the second policy in 2020, hospital-based HaH beds decreased (β1 + β3 + β9 = −76.500, P < 0.01).

**Conclusion:**

While policy reforms expanded HaH service capacity, challenges remain in sustaining home-based medical visits and ensuring workforce supply. Future policies should prioritize multidisciplinary care teams and address barriers to home-based geriatric healthcare to meet the needs of China’s aging population.

